# Case of Erysipelas, Rheumatism, Jaundice, and Abortion, Followed by Puerperal Fever and Death

**Published:** 1846-11

**Authors:** J. Crawford

**Affiliations:** Lecturer on Clinical Medicine and Surgery, M’Gill College, &c.


*Case of Erysipelas, Rheumatism, Jaundice, and Abortion, followed by Puerperal Fever and Death.* By J. Crawford, lecturer on Clinical Medicine and Surgery, M'Gill College, &c.—Mary French,

ætatis 19, a Canadian, unmarried, of spare figure, dark sallow complexion, and bilious temperament, was admitted into the Montreal General Hospital, (18th October, 1845) for an extensive erysipelatous eruption over the right arm, elbow and forearm, which she has had for six days. About two or three days previous to the appearance of the erysipelas, her right elbow and right knee were effected by rheumatic pains, which subsided on the appearance of the exanthem; and she has not had any pain since, unless what may be attributed to the erysipelas ; she has also been affected by jaundice for about the same length of time. She had not any thing done for her complaints previously to her admission ; at which time her right arm and forearm were considerably swelled, and covered by a bright erysipelatous eruption. The adnata of her eyes was very yellow, and her skin generally tinged of the same hue ; her urine was also deeply coloured. The limb was stiff and painful, but nothing to compare with the pain she had suffered at first. She had also smart febrile symptoms, her pulse 108, full, tongue foul, with nausea, hot dry skin, and thirst. She was ordered a purgative of jalap and calomel, and her arm was directed to be brushed over with the tincture of iodine. 20th. The limb is much less swollen, and the redness is paler, and has not extended any farther; there is, however, a great deal of anxiety of countenance, and indication of bodily suffering; her bowels are freely open by the purgative, and she has been taking calomel and Dover's powder four times a day; she is also ordered the infusion of senna to keep up an action in her bowels. 25th. The erysipelas has gradually been subsiding since last report, and is now much better. A small tumour has made its appearance a few days ago, on the inner side of the right elbow, which feels as if there were a collection of matter formed; it is, however decreasing, and appears as if it would be dispersed by the tincture of iodine, which has daily been applied to it. There is still considerable febrile disturbance, with flushing and anxiety of countenance, and profuse acid perspiration, particularly at night: and she complains since last night of rheumatic pain of her right knee, and of her left wrist, which are both slightly swelled ; there is no abnormal sound from the heart. The icteric colour of the adnata and skin generally, is still very marked ; she slept but little last night, from the pains ; her bowels are free. She continues the calomel and Dover powder, and the application of the tincture of iodine, and in addition is ordered an anodyne draught at night. 28th. The erysipelas nearly gone; the right elbow is affected by severe rheumatic pain, and is very powerless. The left wrist is much easier; there is a small soft tumour at the carpal extremity of the left radius, apparently containing matter. The right knee is still painful, pulse 132, febrile symptoms rather less. In addition to the medicines she was using, she was ordered also nitrate of potass ʒvi. in barley water lbij, to be taken during the 24 hours.

November 2d. She is reported better, the pains much easier, and the erysipelas gone ; the jaundice much as formerly.

7th. The pain of the wrist, and tumour of the radius both less; rather more pain of the right elbow and knee, the forearm mottled blue and yellow, as if the limb had been bruised; slept better ; she continued her medicine; no mercurial effect from the calomel. From this time her complaints became considerably aggravated; her sleep was quite interrupted ; she took a grain of opium every two hours, without any effect ; her stomach became irritable, and she threw up bile; the nitre was discontinued, as it probably had disagreed with the stomach; the infusion of senna was ordered, and the opium to be continued in grain doses every hour ; poppy fomentations to the painful parts. From this she appeared to derive relief ; she slept better, and she could bear to move the affected limbs. There was still occasional bilious vomiting ; she took from 8 to 12 grains of opium in the 24 hours; her bowels were kept open by the infusion of senna; the calomel has been omitted for some days. 18th. She has been tolerably easy since last report; this morning it was stated that she had a miscarriage in the night, the fœtus being about four months, of which condition he had no suspicion. There was now a good deal of febrile excitement, pulse 120 small; the rheumatic pains trifling. Next day there was abdominal pain, augmented by pressure, the febrile symptoms persisting. Ordered fomentations to the abdomen, by means of a bag of bran wrung out of hot water, and of ricini ℥i. cum. tr. opii ʒi. These means afforded only very temporary relief, and she passed a restless night, and raved much ; pulse 130 small, and not hard; abdomen very tender. Ordered to be cupped on the abdomen, and to have a blister to the nape of the neck. These remedies produced very little effect. She became wayward and uncontrollable ; her countenance and conduct indicated mental alienation; her pupils were dilated ; pulse 144 ; tongue clean ; her abdomen having been blistered on the previous day, it cannot be ascertained how far the internal pain is better. From this time she appeared to improve a little; her countenance more natural; she did not complain so much of her abdomen; lay on her side, and moved her limbs freely ; she, however, was constantly desirous to leave her bed ; pulse 144 hard, bowels free. Ordered Tr. digitalis M. viij., and antimon. tartar. gr. $\frac{1}{8}$ in aqua cinnam., ℥i. omni hora. On the 24th she is reported to have slept well during the night, and was much better, and more at ease. She moved her limbs freely; her bowels, still tender on pressure, were freely open; the dejections dark and bilious ; pulse 132, small and not so hard. Was ordered to repeat the blister, and to take calomel and opium four times a day. The following day she was much worse, and seemed very low; her pulse rapid, but still of tolerable volume; had passed a bad night, and seemed to complain of abdominal pain on pressure, but it could not be ascertained whether this was not owing to the effects of the blister. She was ordered to be again cupped on the abdomen. She died next day, after I had left Montreal for England. No *post mortem* inspection was made of the body.

Remarks.—Although erysipelas is usually, if not uniformly, accompanied by a derangement of the biliary function, of which we have in most cases sufficient indication, in the discoloration of the albuginea, and the state of the dejection, I have never seen so obstinate a case of jaundice associated with erysipelas, upon which a long perseverance in the use of mercurials and purgatives did not seem to produce any very decided effect. The association of erysipelas, and other exanthemata, with rheumatism, has been noticed by Dr. Todd, and some other modern writers, and of which I have met a few of these complications. Two other cases of erysipelas and rheumatism occurred in the hospital, about the time the above case was under treatment. The other eruptive diseases which I have seen associated with rheumatism were scarlatina, roseola, and erythema nodosum. Dr. Todd is of opinion that rheumatism, as well as these exanthemata, depended on some morbid alteration of the blood. His views appear to be favoured by some more recent investigations, and may probably eventually be generally adopted. This association (although, in some cases, it materially complicates and aggravates the case) does not interfere with the appropriate treatment of each. When, however, the three affections become combined, the case then becomes of a very serious nature; and when abortion and puerperal fever become superadded, the prognosis is extremely unfavourable.

There was a further peculiarity in this case, namely, the rare formation of matter, as a conseqnence of rheumatism ; its absorption, I think, may fairly be attributed to the effects of the iodine. A question suggests itself, did the abortion arise from the rheumatism seizing on the uterus ? I think we may fairly admit this to be the case, as no other satisfactory cause offers in explanation. She had not been taking any drastic medicine, nor was there any particular aggravation of her complaints at the time.

It is to be regretted that a *post mortem* inspection was not made, as much pathological information might be expected to result therefrom.—*Brit. Amer. Journ. of Med. and Phys. Science.*

